# Comparative Genome-Wide Alternative Splicing Analysis between Preadipocytes and Adipocytes

**DOI:** 10.3390/genes15050640

**Published:** 2024-05-18

**Authors:** Zhongyi Hou, Xin Li, Maosheng Xu, Shengbo Meng, Huifen Xu, Ming Li, Hanfang Cai

**Affiliations:** College of Animal Science and Technology, Henan Agriculture University, Zhengzhou 450046, China; hzy15638521652@163.com (Z.H.); 18848897291@163.com (X.L.); 15136149747@163.com (M.X.); 15890651508@163.com (S.M.); huifen221@126.com (H.X.); 13803849306@163.com (M.L.)

**Keywords:** cattle, fat deposition, preadipocytes, adipocytes, RNA-seq

## Abstract

Alternative splicing is a ubiquitous regulatory mechanism in gene expression that allows a single gene to generate multiple messenger RNAs (mRNAs). Adipocyte development is regulated by many processes, and recent studies have found that splicing factors also play an important role in adipogenic development. In the present study, we further investigated the differences in selective shearing during different periods of adipocyte differentiation. We identified five alternative splicing types including skipped exon, mutually exclusive exon, Alternative 5′ splice site, Alternative 3′ splice site, and Retained intron, with skipped exons being the most abundant type of selective shearing. In total, 641 differentially expressed selective shearing genes were obtained, enriched in 279 pathways, from which we selected and verified the accuracy of the sequencing results. Overall, RNA-seq revealed changes in the splicing and expression levels of these new candidate genes between precursor adipocytes and adipocytes, suggesting that they may be involved in adipocyte generation and differentiation.

## 1. Introduction

Fat is widely distributed in various tissues of the organism and plays an important role in the growth and development of animals. The main role of fat is to store and provide energy for the body, maintain the balance of the body’s energy metabolism, regulate the endocrine system, and fix and protect tissues and organs [1]. The adipose tissue of animals includes subcutaneous fat, visceral fat, intermuscular fat, and intramuscular fat.

Intramuscular fat (IMF) is a large accumulation of fat in the muscle, which is mainly distributed in the epimysium, fasciculus, and endomysium. Intramuscular fat has many physiological functions: firstly, it provides energy for the body; and secondly, it can dissolve small-molecule compounds into nutrients that provide energy for the body. Studies have shown that intramuscular fat content has an effect on meat quality in terms of shear, tenderness, flavor, and sensory scores [2]. Beef is favored by consumers because it is rich in protein and amino acids, and low in cholesterol. The higher the intramuscular fat content, the better the quality of the beef. Beef with a rich marbling pattern is called “snowflake beef” and is often categorized as superior meat. Increased intramuscular fat content can improve beef quality and bring higher economic benefits to the beef market. Therefore, analyzing the molecular regulatory mechanisms of animal fat deposition and improving meat quality are important research directions for the development of animal husbandry.

There is growing evidence that epigenetic modifications play an important role in the regulation of adipogenesis [3,4]. Epigenetic regulation, which includes DNA methylation [5], RNA methylation [6], histone modifications [7], and noncoding RNAs [8], is a genetic mechanism that alters gene expression and function without altering the DNA sequence.

Differential splicing, also known as alternative splicing, is a type of ribonucleic acid (RNA) processing in organisms. It allows a gene to be spliced in different ways to produce a variety of different types of mature mRNA, which in turn translates into a variety of different proteins [9]. Existing findings suggest that alternative splicing is a pervasive regulatory mechanism during gene expression and plays an important role in many biological processes, such as cell differentiation and development [10], immune response [11], and apoptosis [12]. At the same time, it provides an important bridge between the genome and proteome [13,14]. Currently available findings emphasize the importance of alternative splicing in various pathologies and normal physiology, mainly in cancer, immunity, and disease, while few studies have focused on livestock genetics and production improvement. An in-depth understanding of the importance of alternative splicing in the economic shape of livestock and its consequent improvement is also an area of interest. Similarly, data on selective shearing in bovine adipocytes have rarely been studied and reported. Xianan cattle is the first beef cattle breed independently bred in China, which has the characteristics of gentle temperament, rough feeding resistance, fast growth, and easy fattening. It is mainly distributed in Biyang County, Henan Province. In this study, we used adipocytes isolated from Xianan cattle as experimental materials, and analyzed the different selective shearing of adipocytes occurring before and after differentiation by RNA-seq, which may provide a new theoretical basis for the identification of transcripts and aid in exploring strategies to improve the quality of beef from the perspective of epigenetic regulation.

## 2. Materials and Methods

### 2.1. Ethics Statement

All experimental procedures were approved by the Institutional Animals Care and Use Committee (IACUC) of the College of Animal Science and Technology of Henan Agricultural University, China (Permit Number: 11-0085; Date: June 2011).

### 2.2. Isolation and Culture of Preadipocytes

Abdominal subcutaneous fat was taken from three Xianan fetal cows of about three months’ size, and the adipose tissue was washed twice with phosphate buffer solution (PBS) containing 2% Penicillin-Streptomycin Solution (Procell, Wuhan, China), and then cut into a mud with scissors. Afterwards, the tissue was added to a digestion solution containing 0.2% Collagenase I (Solarbio, Beijing, China) and placed in a 37 °C water bath to digest the tissue into a flocculent mass.

After digestion, the cells were filtered through a 40 mesh sieve, a 200 mesh sieve, and a 400 mesh sieve in order to remove undigested tissues. After centrifugation at 1000 rpm for 5 min, the cells were resuspended in DMEM/F12 medium containing 10% fetal bovine serum (FBS) and %1 Penicillin-Streptomycin Solution and inoculated at a density of approximately 1 × 10^6^ cells into 25 cm^2^ culture flasks. After adding complete medium (DMEM/F12 containing 10% FBS and 1% Penicillin-Streptomycin Solution) to the culture flasks, the flasks were incubated at 37° in an incubator containing 5% CO_2_.

### 2.3. Induced Differentiation of Preadipocytes

Cells were at 100% density and induction of differentiation was initiated after 48 h of contact inhibition by treating the cells with complete medium containing 1 μmol/L dexamethasone, 0.5 mM 3-isobutyl-1-methylxanthine, and 10 μg/mL insulin. On the second day of induction and differentiation, the induction medium was replaced with induction maintenance medium (DMEM/F12 containing 10% fetal bovine serum, 1% Penicillin-Streptomycin Solution and 10 µg/mL insulin). Fresh induction maintenance medium was replaced every 2 days thereafter.

### 2.4. RNA Extraction, Quality Analysis, and Library Construction

RNA was extracted from three replicates each of preadipocytes and adipocytes on the sixth day after differentiation using Trizol reagent (TransGen, Beijing, China), followed by agarose gel electrophoresis to analyze the RNA integrity of the samples and the presence of DNA contamination, and an Agilent 2100 bioanalyzer (Agilent 2100 bioanalyzer (Agilent, Beijing, China) was used to detect the quality and concentration of RNA.

RNA-seq library construction was performed by Novogene (Novogene, Beijing, China). Strand-specific libraries were constructed using the method of removing ribosomal RNA. First, ribosomal RNA was removed from total RNA, and subsequently the RNA was broken into short fragments of 250–300 bp. The fragmented RNA was used as a template, and random oligonucleotides were used as primers to synthesize the first strand of cDNA, followed by the second strand of cDNA with dNTPs (dUTP, dATP, dGTP, and dCTP). The purified double-stranded cDNA was subjected to end repair, addition of A-tail and ligation of sequencing junction, and screened for cDNAs around 350~400 bp using AMPure XP beads. cDNA second strand containing U was degraded using USER enzyme (New England Biolabs, Ipswich, MA, USA), and finally PCR amplification was carried out and the library was obtained.

### 2.5. Variable Splicing Analysis

The results were categorized for alternative splicing (AS) and analyzed for differential AS using the rMATS (4.1.2) software comparison. The software can categorize AS events into 5 categories and allows differential AS analysis of samples with biological replicates. Each variable clipping event corresponds to two Isoforms, the Exon Inclusion Isoform and the Exon Skipping Isoform. Isoforms were counted and divided by their effective lengths to obtain the corrected expression, and then the ratio of the total expression of Exon Inclusion Isoform to that of Exon Skipping Isoform was calculated, and finally the significance of difference was analyzed.

### 2.6. Gene Ontology and Kyoto Encyclopedia of Genes and Genomics Enrichment Analysis

Gene Ontology (GO) annotation was performed using Omicshare online software to analyze the annotation function of differentially selective shear genes. Differential selective shear genes were then analyzed for pathway analysis using Omicshare online software. Data were generated using OmicShare online website (http://www.omicshare.com/tools accessed on 20 November 2023).

### 2.7. Identification of Differentially Alternative Splicing Transcripts

One microgram of total RNA cDNA was synthesized using HiScript II Q RT SuperMix for qPCR (Vazyme, Nanjing, China). Primers were designed using rimer Premier 6.0 software (Premier BioSoft, Palo Alto, CA, USA). The PCR reaction system was 50 ul, including 25 µL of 2× Rapid Taq Master Mix (Vazyme, Nanjing, China), 2 µL of Forward primer and Reverse prime (10 µM), 2 µL of cDNA and 19 µL of ultrapure water. The PCR reaction program was as follows: 95 °C pre-denaturation for 3 min; followed by 35 cycles of 95 °C for 15 s, 60 °C for 15 s, and 72 °C for 5 s; and finally, a complete extension at 72 °C for 5 min. The PCR reaction products were later detected on a 1% agarose gel. Primers for PCR can be found in the Supplementary Material Table S1.

### 2.8. Quantitative Real-Time PCR

Real-time fluorescence quantification of mRNA expression levels of differentially expressed alternative splicing genes in preadipocytes and adipocytes using the CFX96 Touch Real-Time PCR Detection System (Bio-Rad, Hercules, CA, USA). The real-time PCR system was as follows: 5 µL 2× ChamQ SYBR qPCR Master Mix (Vazyme, Nanjing, China), 2 µL cDNA, 0.2 µL Forward primer (10 µM), 0.2 µL Reverse prime (10 µM), and 2.6 µL RNase-free water. The reaction program was as follows: 95 °C for 30 s followed by 40 cycles: 95 °C for 10 s and 60 °C for 30 s. Primers for RT-qPCR can be found in the Supplementary Material Table S1.

Relative expression levels were calculated using 2^−(∆∆Ct)^ using β-Actin as the reference gene. Data are available as mean ± SEM of at least three independent experiments. Statistical analyses were performed using independent samples *t*-tests (*n* = 3) with significance levels of *p* < 0.05 (*), *p* < 0.01 (**), *p* < 0.001 (***).

## 3. Results

### 3.1. RNA-Seq and Transcriptome Analysis of Preadipocytes and Adipocytes

A total of 507,348,102 raw reads were obtained from six samples of preadipocytes and adipocytes. After raw data filtering, sequencing error rate checking and CG content distribution, a total of 494,201,012 clean reads were obtained. The clean reads obtained from sequencing were compared to the reference genome Bos taurus (UMD_3.1.1), which can be compared to a single position of the genome with a READS number between 89.22% and 91.73%. The quality of sequencing data is detailed in Table 1.

**Figure 1:**
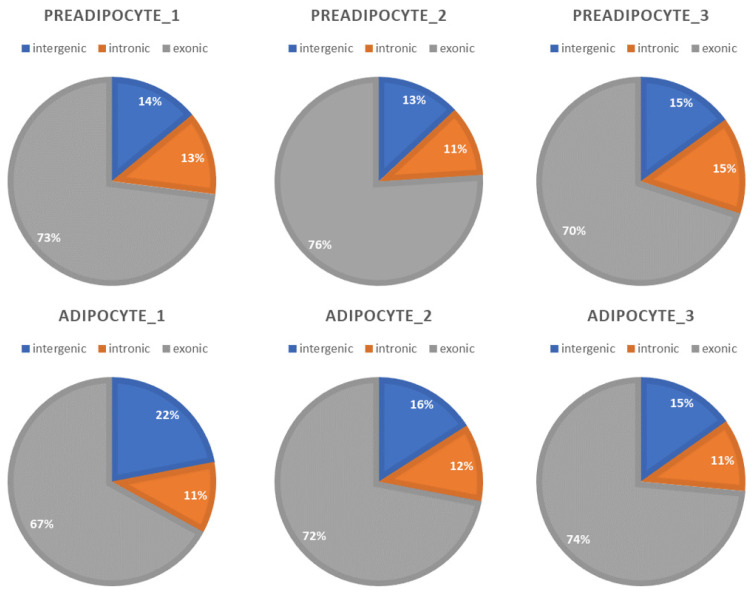
Percent of mapped to genome regions.

Figure 1 shows the proportion mapped to exonic regions, intronic regions, and intergenic regions of the genome.

### 3.2. Forms and Expression of Alternative Splicing

**Figure 2:**
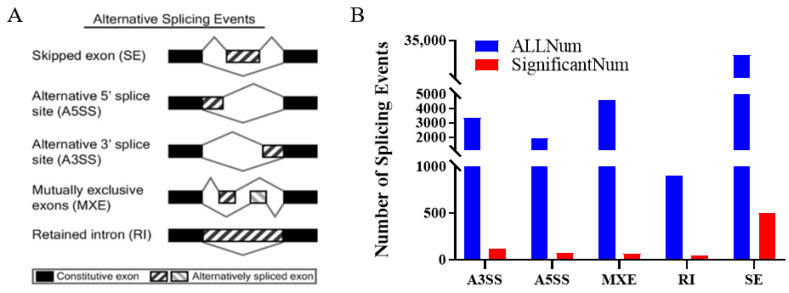
Differentially expressed alternatively spliced genes analysis. (**A**) Alternative splicing types. (**B**) Number of alternative splicing types. The black blocks represent constitutive exons, and the white blocks represent alternatively spliced exons.

A total of 40,052 alternative splicing events corresponding to 9369 genes were identified through differential alternative splicing analysis. These included five forms of alternative splicing: skipped exon (SE), mutually exclusive exon (MXE), alternative 5′ splice site (A5SS), alternative 3′ splice site (A3SS), and retained intron (RI) (Figure 2A). The number of alternative splicing events occurring in descending order was SE, A3SS, A5SS, MXE and RI.

Based on the adjusted *p*-value (*p* < 0.05), 804 differentially expressed alternative splicing events were screened, including 499 SE, 118 A3SS, 73 A5SS, 66 MXE and 48 RI (Figure 2B). A total of 641 genes corresponding to 804 differentially expressed alternative splicing events were identified, including *CD47*, *TPM1*, *HNRNPC*, *POLR2D*, *IRAK4*, etc. The differentially expressed alternative splicing genes are detailed in Table 2. All 641 alternative splicing difference genes can be found in the Supplementary Material Table S2.

### 3.3. GO and KEGG Enrichment of Differentially Expressed Alternatively Spliced Genes

A total of 641 differentially expressed alternative splicing genes were identified using a *p*-value < 0.05 and |fold change| > 2 as the screening criteria. In all, 483 GO terms were found to be significantly enriched in the bioprocesses by GO functional enrichment analysis of the differentially expressed alternative splicing genes. Among these, organelle organization (GO:0006996), cellular component organization or biogenesis (GO:0071840) and cellular component organization (GO:0016043) are a few of the most significantly enriched GO terms. A total of 136 GO terms were significantly enriched in molecular functions, binding (GO:0005488), protein binding (GO:0005515), and torganic cyclic compound binding (GO:0097159) being a few of the most significantly enriched GO terms. Similarly, 120 GO terms were significantly enriched in cellular components, intracellular anatomical structure (GO:0005622), intracellular organelle (GO:0043229), and organelle (GO:0043226) being some of the most significantly enriched GO terms. The top 25 GO terms for biological processes, molecular functions, and cellular components are listed in ascending order of *p*-value.

**Figure 3:**
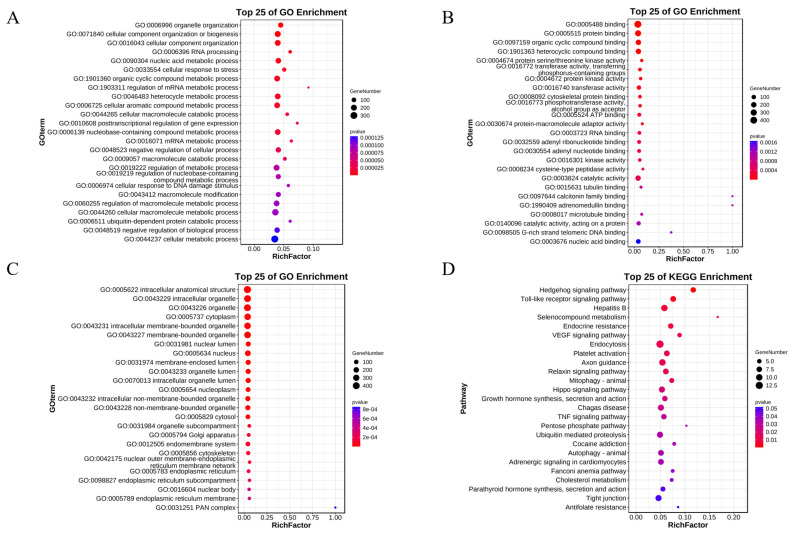
Gene Ontology (GO) and Kyoto Encyclopedia of Genes and Genomes (KEGG) enrichment. (**A**–**C**) GO enrichment analysis of biological processes, molecular functions, and cellular components. (**D**) KEGG enrichment of differentially expressed genes.

Subsequent KEGG functional enrichment analysis of the 641 differentially expressed variable shear genes revealed enrichment in 279 pathways, with a total of 24 pathways significantly enriched (*p* < 0.05). These included the Hedgehog signaling pathway (PATH: 05161), Selenocompound metabolism (PATH: 00450), and Endocrine resistance (PATH: 01522). As shown in Figure 3B, the top 25 enriched pathways are listed according to *p*-value from low to high.

### 3.4. Identification of Alternative Splicing Types and Verification of Expression Levels of Transcripts

**Figure 4:**
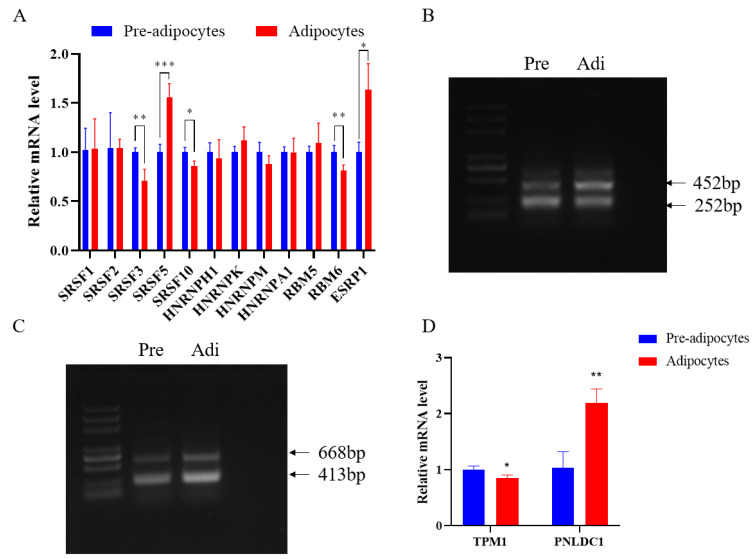
Verification of splicing factor, splicing type, and expression level. (**A**) Relative expression levels of splicing factor. (**B**) PCR validation of *TPM1* (RI). (**C**) PCR validation of *PNLDC1* (RI). (**D**) Relative expression levels of *TPM1* and *PNLDC1*. *p* < 0.05 (*), *p* < 0.01 (**), *p* < 0.001 (***).

To further validate the accuracy of the sequencing and prediction data, we examined the expression of the alternative splicing factor SRSF gene family, RBM gene family and HNRNP gene family before and after adipocyte differentiation. Significant differences were found in the *SRSF3*, *SRSF5*, *SRSF10*, *RBM6* and *ESRP1* genes (Figure 4A). Following this, we validated tropomyosin 1 (*TPM1*) and PARN like ribonuclease domain containing exonuclease 1 (*PNLDC1*) by PCR and RT-qPCR. *TPM1* has three transcripts, the long transcript is expressed at a lower level in pre-adipocytes, and *PNLDC1* also has two transcripts, both of which are expressed at a higher level in adipocytes. (Figure 4C). RT-qPCR results showed that the expression of *TPM1* decreased after adipocyte differentiation, and the expression of *PNLDC1* increased after adipocyte differentiation. The results show that, consistent with the sequencing results, *TPM1* and *PNLDC1* incidence differential variable shearing during adipocyte differentiation.

## 4. Discussion

In the present study, 40,052 selective shearing events corresponding to 9369 genes indicate that selective shearing genes are prevalent before and after adipocyte differentiation, with skipped exons being the most abundant type of selective shearing, similar to that reported in other livestock animals [15,16,17].

Splicing factors play an important role in adipogenesis [18,19], several studies have investigated the relationship between alternative splicing and adipogenesis, with variants encoded by variably sheared transcripts having different or even opposing effects on adipogenesis. The splicing factor *SRSF10* is an atypical SR protein that can function as a sequence-dependent shear activator by directly binding to exonic shear enhancers [20]. Lipin1 plays a key role in glycerol ester biosynthesis and transcriptional coactivators, and in adipocyte development and maturation [21], and deletion of *SRSF10* leads to lipin1 precursor mRNA shear changes thereby leading to lipogenesis defects [22]. *SF3B1* is a key component of the spliceosome [23] and a key factor in the normal thermal activation of differentiated brown adipocytes [24]. It can mediate the expression pattern of selective splice genes thereby enhancing thermogenic activity. *RBM4* and *SRSF3* can regulate brown adipogenesis with the *MAPK4* shear cascade through related signaling pathways [25].

Tropomyosins (TPM) are a family of actin-related proteins associated with the pathology of neurodegenerative and neurological diseases [26], and *TPM1* is a widely expressed actin-binding protein in the TPM family. Some findings suggest that *TPM1* plays a previously unexpected role in regulating pro-inflammatory genes in microglia, in addition to its critical role in regulating neuronal synapse growth, branching, and synapse formation. *TPM1* can promote LPS-mediated inflammation and peripheral neuronal cell death in microglia through the PKA/CREB pathway [27]. Tropomyosin 1 (*TPM1*) plays an important role in lung cancer, colorectal cancer, and gastric cancer [28,29]. Recently, it has been found that m6A modification of *TPM1* can promote the therapeutic efficacy of prostate cancer by mediating m6A modification [30], which affects the expression level of mRNA by affecting its shearing and translation [31]. Whether the differential shearing of *TPM1* is regulated by m6A modification needs to be further verified. *PNLDC1* is a novel poly(A)-specific nucleic acid exonuclease that has been found to be discretely expressed during early mammalian development. In mouse embryonic stem cells, it is expressed only during early development and gradually decreases after differentiation due to epigenetic events [32]. The results of the present study indicate that the expression of *PNLDC1* gradually increases during adipocyte development, but the function of *PNLDC1* in adipocyte development needs to be further verified.

The current study found that many genes have multiple transcripts with different levels of expression, the expression levels of different transcripts of the same gene need to be further investigated for specific genetic functions and regulatory mechanisms for bovine adipocyte development and fat deposition and meat quality traits. Whether alternative splicing is dependent on some epigenetic modifications, such as m6A modification, for its role in life activities is also worth exploring.

## 5. Conclusions

In the present study, transcriptome analysis by RNA-seq identified 641 genes that underwent significant differential alternative splicing events before and after adipocyte differentiation. Both *TPM1* and *PNLDC1* undergo differential alternative splicing events before and after adipocyte differentiation. The long transcript of *TPM1* was expressed at low levels in pre-differentiated adipocytes, and both *PNLDC1* transcripts were expressed at high levels in post-differentiated adipocytes. Additionally, the expression levels of several splicing factors were verified before and after adipocyte differentiation. It was found that the mRNA expression levels of *SRSF5* and *ESRP1* were significantly increased after adipocyte differentiation. *SRSF3*, *SRSF10*, and *RBM6* were significantly reduced after adipocyte differentiation.

## Figures and Tables

**Table 1:** Details of sequencing data by RNA-seq.

Samples	Total Reads	Clean Reads	Unique Mapped	Reads Filter %	Unique Mapped Rat %
Pre-1	82,899,462	81,014,722	74,318,042	97.73%	91.73%
Pre-2	91,252,432	89,623,528	80,020,163	98.21%	89.28%
Pre-3	83,152,664	80,554,612	72,714,018	96.88%	90.27%
Adi-1	83,587,552	80,441,578	72,794,729	96.24%	90.49%
Adi-2	79,558,574	77,525,064	69,168,973	97.44%	89.22%
Adi-3	86,897,418	85,041,508	77,119,851	97.86%	90.68%

**Table 2:** Top 15 differentially expressed alternatively spliced genes between preadipocyte and adipocyte.

Gene ID	Location	Adi Exp	Pre Exp	*p*-Value	FDR	Splicing Type
*CD47*	chr1	11::2291	136::1943	0	0	SE
*HNRNPC*	chr10	313::1	206::19	0	0	SE
*IRAK4*	chr5	391::32	403::0	0	0	SE
*CD200*	chr1	48::0	34::17	0	0	SE
*VCAN*	chr7	525::668	8074::463	0	0	SE
*POLR2D*	chr2	1172::37	1338::1	0	0	SE
*FBLN2*	chr22	34::457	234::864	0	0	SE
*CD200*	chr1	3::49	46::37	0	0	MXE
*CSNK1G1*	chr10	1::14	22::6	0	0	MXE
*HNRNPC*	chr10	117::1	94::19	8.88 × 10^−16^	3.25 × 10^−12^	SE
*SNRPA1*	chr21	207::28	466::9	9.99 × 10^−16^	3.25 × 10^−12^	SE
*RTCA*	chr3	5::15	11::0	1.89 × 10^−15^	6.38 × 10^−12^	A3SS
*POLQ*	chr1	3::0	1::14	3.00 × 10^−15^	8.76 × 10^−12^	SE
*CCND3*	chr23	693::49	394::3	3.77 × 10^−15^	1.00 × 10^−11^	SE
*SGMS1*	chr26	2::7	5::0	4.88 × 10^−15^	1.19 × 10^−11^	SE

## Data Availability

The original contributions presented in the study are included in the article/Supplementary Material, further inquiries can be directed to the corresponding author.

## Supplementary Materials

The following supporting information can be downloaded at: https://www.mdpi.com/article/10.3390/genes15050640/s1, Table S1: Primers for RT-qPCR; Table S2: All 641 alternative splicing difference genes.
