# Supplementary material for: Comparative Genome-Wide Alternative Splicing Analysis between Preadipocytes and Adipocytes
Source: Genes (Basel). 2024 May 18;15(5):640. doi: 10.3390/genes15050640 (PMC11121090; doi:10.3390/genes15050640)
Supplement: Supplementary file 1 [file genes-15-00640-s001.zip › Table S1. Primer.pdf]

Primers of RT-PCR

TPM1
F: CGTCGGTGGCCTTTTTCTCCGC
R: CTCGAGGAGGACATAGCGGCCA
PNLDC1-F
F: GCGCTGTTCCCATCCCCGAG
R: GAGCAGGAACTGGCTCCGCG

Primers of RT-qPCR

SRSF1
F: GATATCGTCTGCGGGTGGAG
R: TGCTTCACGCATGTGATCCT
SRSF2
F: CTAACCTACCGCACCTCACC
R: GACTCCTTGGTGTAGCGGTC
SRSF3
F: ACTCCGAAGTGTGTGGGTTG
R: TTCCACTCTTACTCGGCAGC
SRSF5
F: GGGATGCGGATGATGCTGTA
R: AAACGGTCTGAGTAGCGTCC
SRSF10
F: TTGCACAGGGGGATCGAAAG
R: TTCCAGTCGGTCTATTTCTAGGAC
HNRNPH1
F: TGCTGTGGCAGCTATGTCAA
R: CTGGTTTGACAAGCCCATGC
HNRNPK
F: AGGAAGACCTGGAGACCGTT
R: CCATCTGCCACTCTGATGGG
HNRNPM
F: CTGAAGGAAAGTCAAGGGGATGT
R: AGTCGTAGCCATCACCTTTTGC
HNRNPA1
F: CGAGGCAGTGGCAAAAAGAG
R: TGGCTGGATGAAGCACTAGC
RBM5
F: AATTACGAGCAGCCCACCAA
R: CTGCACCAGACAGTCCGTAT
RBM6
F: ACCTTCCGAAGAGACCGAGA
R: GTAAGGCGGACATAGGGCTC

ESRP1

F: AAATGGCTTATCCCCACCGC

R: ACTGAGGGCTGGTAAATGGC
